# Intake of a fermented plant product attenuates allergic symptoms without changing systemic immune responses in a mouse model of Japanese cedar pollinosis

**DOI:** 10.1186/s40413-018-0213-4

**Published:** 2018-12-04

**Authors:** Takashi Fujimura, Ayane Hori, Hideto Torii, Shinsuke Kishida, Yoshinori Matsuura, Seiji Kawamoto

**Affiliations:** 1 Hiroshima Research Center for Healthy Aging (HiHA), Graduate School of Advanced Sciences of Matter, Hiroshima University, 1-3-1 Kagamiyama Higashi-Hiroshima, Hiroshima, 739-8530 Japan; 2 Manda Fermentation Co., Ltd, 5800-95 Innoshima, Shigei, Onomichi, Hiroshima, 722-2192 Japan

**Keywords:** Allergy, Fermented plant product, Japanese cedar, Pollinosis, Rhinitis

## Abstract

**Background:**

Japanese cedar pollinosis (JCP) is one of the most prevalent allergies in Japan. Within the past few decades, many food factors have been demonstrated to suppress symptoms of pollinosis and mast cell degranulation directly or indirectly. Herein, we conducted a study to clarify the anti-allergic potency of a fermented plant product (FPP) in JCP model mice.

**Methods:**

Mice were administered FPP, 10-fold-diluted FPP, or saline every day for 40 days by oral gavage and sensitized with major Japanese cedar pollen allergens (SBP). The numbers of sneezes were counted for 5 minutes after SBP nasal challenge. We analyzed the SBP-specific immunoglobulin titers, serum concentration of mast cell protease 1, and cytokine production from splenocytes stimulated with SBP.

**Results:**

The numbers of sneezes by the mice administered FPP were significantly suppressed compared to those administered saline. The 10-fold-diluted FPP also suppressed the number of sneezes compared to saline, although not significantly. Serum level of mast cell protease 1 tended to be suppressed in FPP-consumed mice compared to those in saline-treated mice. The SBP-specific immunoglobulin titers and cytokine production were comparable among the groups.

**Conclusions:**

Our results suggest that FPP intake could attenuate JCP symptoms without change of systemic immune responses.

## Background

Japanese cedar (*Cryptomeria japonica*) pollen is one of the major causes of seasonal allergic rhinitis, and the increase of patients suffering Japanese cedar pollinosis (JCP) has been a severe social problem in Japan [1]. Since the first report of JCP’s appearance in 1964 at Nikko area of Tochigi prefecture in Japan, its prevalence has steadily increased, and a nationwide survey showed that the prevalence of JCP doubled from 13.1% in 2001 to 26.5% in 2008 [2–4]. The first major Japanese cedar pollen allergens to be reported are a pectate lyase (Cry j 1) and a polygalacturonase (Cry j 2), and another eight allergens were later characterized [1]. The allergens Cry j 1 and Cry j 2 are also important as major ingredients of a curative vaccine for allergen-specific immunotherapy against JCP [5–7].

Pollinosis is triggered by an invasion of pollen grains onto nasal and ocular mucosa, followed by a release of allergens from pollen grains onto the aqueous phase of the mucosal membrane. The released allergens cross-link allergen-specific immunoglobulin E (IgE) bound on surface high-affinity IgE receptors (FcɛRI) of mast cells. The cross-linking of IgEs on mast cell triggers the release of inflammatory chemical mediators including histamine that induce clinical symptoms such as sneezing and itching [1, 8].

Suppression of mast cell degranulation is crucial to attenuate IgE-mediated allergic symptoms including asthma, rhinitis, conjunctivitis, and atopic dermatitis [9]. Not only direct suppression by chemicals, but also neutralization of allergens by allergen-specific immunoglobulins can prevent IgE-mediated mast cell degranulation [6, 10–12]. Amelioration of allergic symptoms by increased IgG and IgG4 antibodies was observed in positive responders of allergen-specific immunotherapy [1, 12]. Allergen-specific immunotherapy can increase the level of allergen-specific IgG and IgA in nasal mucosa and these immunoglobulins prevent binding between allergen and allergen-specific IgE in nasal cavity. These interruptions of binding of allergens and allergen-specific IgE prevent following IgE-dependent mast cell degranulation, and thus ameliorate allergic symptoms [13].

Within the past few decades, many food factors have been demonstrated to suppress Th2-type responses and mast cell degranulation directly or indirectly [10, 14–16]. It was recently reported that fermented *Glycine max* ameliorates atopic inflammations in skin, accompanied by a suppression of protein kinase C and the production of interleukin (IL)4, a Th2-type cytokine, in atopic dermatitis-prone NC/Nga mice [17]. In a clinical study conducted in Korea, the intake of fermented food is associated with a low prevalence of atopic dermatitis [18]. Fermented products – especially fermented plants including vegetables and fruits – are used as anti-inflammatory and anti-allergic medicines, as are Chinese medicinal herbs.

Fermented plant product (FPP) is a fermented supplemental food made from a variety of fruits, citrus, root crops, grains, pulses, marine algae and raw cane sugar fermented for > 3 years + 3 months at room temperature [19, 20]. The ability of FPP to improve animal or human health by oral consumption has been described. For example, the consumption of FPP was reported to improve the emotional stress-induced stomach ulcers and age-related neuronal damage by oxidative stress in rats [21, 22]. In Japanese flounder (*Paralichthys olivacus*), the consumption of FPP activates innate immunity and prevents hemolysis and lipid peroxidation, and in mice FPP consumption showed carcinostatic effects on adaptive transferred tumor cells by its high anti-oxidant capacity [20, 23, 24]. Carcinostatic effects of FPP on human breast cancer cells were also demonstrated [25]. The key mechanism of FPP’s health-improving capacity is ascribed to its strong anti-oxidative capacity [22, 23]. However, the effects of consumption of FPP on the progression of allergic inflammation have not been determined. In this study, we used a JCP mouse model to analyze the ability of FPP to ameliorate clinical symptoms of seasonal allergic rhinitis.

## Methods

### Fermented plant product and chemicals

Fermented plant product (FPP, MANDA®) was provided by Manda Fermentation Co., Ltd. (Onomichi, Japan). FPP was made from a variety of fruits (26.1%), citrus (14.0%), root crops (5.3%), grains (8.1%), pulses (5.2%), marine algae (5.3%) and raw cane sugar (33.4%) fermented with unidentified microbes including yeasts and *Lactobacillus* for > 3 years + 3 months at room temperature [19, 20]. The FPP is a highly viscous black-color fermented foodstuff containing 2.2% protein, 0.001% lipid, 60.3% carbohydrate, 2.6% food fiber, 1.9% ash, 32.9% water, and several minerals and vitamins [19, 22]. FPP was kept at room temperature and protected from light. All of the chemicals used were of biochemical grade or cell-culture grade, and were purchased from Wako Pure Chemical Industries (Osaka, Japan) unless otherwise indicated.

### Preparation of Sugi basic protein

Sugi basic protein (SBP), a mixture of the major Japanese cedar pollen allergens Cry j 1 and Cry j 2, was prepared as described with slight modification [26, 27]. Briefly, 40 g of Japanese cedar (*C. japonica*) pollen was suspended in 125 mM sodium bicarbonate buffer (pH 8.0) containing 3 mM ethylenediamine tetraacetic acid overnight at 4 °C, and the suspension was centrifuged at 7,400 *g* for 35 minutes at 4 °C. Ammonium sulfate was added to the supernatant until 80% saturation, and the solution was stirred overnight at 4 °C. The resultant precipitate was dialyzed against 5 mM phosphate buffer (pH 7.5) and then applied directly to a DEAE-Toyopearl 650 column (Tosoh, Tokyo, Japan). The unadsorbed fraction was applied onto a Micro-Prep® Ceramic Hydroxyapatite type II column (BioRad Laboratories, Hercules, CA, USA), and the adsorbed fraction was obtained by gradient elution from 0 to 0.6 M sodium chloride in 5 mM phosphate buffer (pH 7.5). The fractions containing approx. 45-kDa proteins (SBP) were pooled and dialyzed against phosphate-buffered saline (PBS) at 4 °C. The protein concentration of resultant SBP was determined by a Qubit protein assay kit (Molecular Probes, Thermo Fisher Scientific, Eugene, OR, USA).

### Mouse model experiment

Six-week-old female BALB/c mice were purchased from Charles River Laboratories Japan (Kanagawa, Japan) and kept under specific pathogen-free conditions. All animal experiments were carried out using protocols approved by the Committee on Animal Experimentation of Hiroshima University, Japan.

**Fig. 1:**
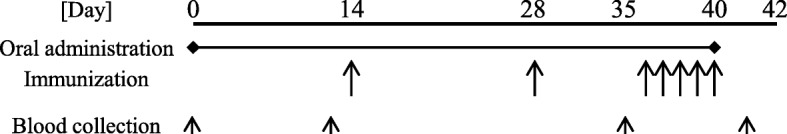
Experimental procedure. Details are given in the Materials and Methods section. One hundred μL of FPP, ten-fold-diluted FPP, or saline was administered orally every day for 40 days. Mice were intraperitoneally immunized with SBP precipitated with alum on day 14, then subcutaneously sensitized with SBP on day 28, and finally intranasally administered SBP every day for 5 days. Peripheral blood was collected at days 0, 13, 35, and 41

The first animal experiment was designed to analyze the capacity of FPP to ameliorate clinical symptoms and to alter SBP-specific antibody titers. The second and third animal experiments were designed to analyze clinical symptoms, SBP-specific antibody titers, and the proliferation of and the cytokine production from murine splenocytes stimulated with SBP. For the three independent experiments, mice were administered 100 μL of FPP, or 10-fold-diluted FPP in endotoxin-free saline (Otsuka Pharmaceutical Factory, Tokushima, Japan), or endotoxin-free saline every day for 40 days by oral gavage (Fig. 1). The mice were intraperitoneally injected with a 5-μg protein weight of SBP with 2 mg of Alum (Alhydrogel; Invivogen, San Diego, CA, USA) in 200 μL of endotoxin-free saline on day 14, and again at 2 weeks after the immunization. The mice were then subcutaneously injected with 5-μg protein weight of SBP on day 28 (Fig. 1). Subsequently, the mice were intranasally administered 10 μL of 100 μg/mL SBP in endotoxin-free saline daily for 5 consecutive days (from day 36 to day 40).

Submandibular blood was collected via a facial vein on days 0, 13, 35 and 41 (Fig. 1). The peripheral blood samples were incubated at 4 °C for 1 hour, then centrifuged at 12,000 *g* for 10 minutes at 4 °C, and sera were obtained. The sera were stored at − 20 °C until analysis. The number of times each mouse sneezed during a 5-minute period soon after an intranasal challenge with SBP was counted. For the 1st, 2nd and 3rd experiments, final numbers of mice were 5, 6, and 5 for FPP; 5, 6, and 5 for 10-fold-diluted FPP; and 6, 12, and 12 for saline, respectively.

### ELISA for SBP-specific IgE, IgG, and IgA and for inhibitory assay of IgE binding

The titers for SBP-specific IgE were determined as described with slight modifications [6]. Briefly, 100 μL of 1 μg/mL monoclonal antibody to mouse IgE (clone 6HD5; Yamasa, Tokyo, Japan) in 0.1 mM bicarbonate buffer (pH 9.5) was applied to 96-well microtiter plates (Nunc-Immuno® Maxisorp, Thermo Fisher Scientific) and incubated at room temperature for 2 hours. The plates were blocked with blocking buffer, i.e., PBS containing 0.5% bovine serum albumin (BSA; Sigma-Aldrich, St Louis, MO, USA), 5% fetal bovine serum (FBS; MP Biomedicals, Solon, OH, USA), 0.02% sodium azide, and 0.05% Tween 20 at room temperature for 1 hour. Then, 100 μL of 100-fold-diluted sera from mice in PBS containing 10% FBS, 0.02% sodium azide, and 0.05% Tween 20 was added to the wells, and the plates were incubated overnight at 4 °C. Then, 100 μL of 0.1 μg/mL biotin-labeled SBP in dilution buffer, i.e., PBS containing 1% BSA and 0.05% Tween 20 was added and the plates were incubated at room temperature for 1 hour.

Next, 100 μL of 5,000-fold-diluted streptavidin-β-galactosidase-conjugate (Roche Diagnostics, Mannheim, Germany) was added to the wells, and the plates were incubated at room temperature for 1 hour. For the enzymatic reaction, 0.2 mM 4-methylumbelliferyl β-D-galactopyranoside (Sigma-Aldrich) was added and the plates were incubated at 37 °C for 2 h. After the reaction was stopped with 0.1 M glycine-NaOH (pH 10.2), we measured the fluorescence intensity using a Wallac 1420 ARVOsx multilabel counter (PerkinElmer Life Sciences, Waltham, MA, USA).

Inhibition ELISA of binding between SBP and SBP-specific IgE by other immunoglobulin isotypes was performed according to the method previously described by using SBP instead of Cry j 1 [6].

For the analysis of SBP-specific IgG and IgA, 1 μg/mL SBP in 0.1 M bicarbonate buffer (pH 9.5) was applied onto 96-well microtiter plates, and the plates were incubated overnight at 4 °C. After the plates were blocked with blocking buffer, 100 μL of 30,000- or 100-fold diluted murine sera was added to the wells for IgG or IgA, respectively, and the plates were incubated at room temperature for 1 h. Then, 100 μL of 2,000-fold diluted affinity-purified antibody peroxidase-labeled goat anti-mouse IgG (KPL, Gaithersburg, MD, USA) for IgG or 1,000-fold-diluted horseradish peroxidase-conjugated goat anti-mouse IgA alpha chain (Abcam, Cambridge, MA, USA) for IgA was added, and the plates were incubated at room temperature for 1 hour.

For the enzymatic reaction, 100 μL of 3,3′,5,5′-tetramethylbenzidine (TMB) enzyme-linked immunosorbent assay (ELISA) substrate solution (eBiosciences, Thermo Fisher Scientific) was added and the plates were incubated at room temperature. After the reaction was stopped by adding 25 μL of 1.0 M sulfuric acid, we measured the absorbance at 450 nm with the Wallac 1420 ARVOsx multilabel counter.

The serum titers of SBP-specific IgE and SBP-specific IgG were calculated on a standard curve generated using reference pooled serum from SBP-sensitized mice as 1 kAU (arbitrary unit)/L.

### ELISA for serum mouse mast cell protease (MMCP)1 of the immunized mice

Serum concentration of MMCP1 in the mice at day 0, 13, 35, and 41 was quantified by a sandwich ELISA using mouse MCPT-1 ELISA kit (Invitrogen, Thermo Fisher Scientific) following the manufacturer’s instructions.

### Cytokine and proliferation assays of splenocytes from immunized mice

First, 5 × 10^5^ splenocytes from sensitized mice were cultured with or without 5 μg/mL SBP or 0.5 μg/mL concanavalin A (Con A) in 200 μL RPMI medium (Sigma-Aldrich) containing 10% FBS (Sigma-Aldrich), 50 μM 2-mercaptoethanol, 100 units/mL penicillin (Thermo Fisher Scientific), and 100 μg/mL streptomycin (Thermo Fisher Scientific) for 3 days. After the culture, BrdU was added to a 10 μM final concentration and the cells were cultured for an additional 18 hours. The BrdU incorporation was determined by a Cell Proliferation Enzyme-linked Immunosorbent Assay BrdU (colorimetric) Kit (Roche Diagnosis) following the manufacturer’s instructions.

For the cytokine assay, plates that had been cultured for 3 days were centrifuged at 1,200 rpm for 5 min at room temperature, and the supernatant was stored at − 20 °C until analysis. The concentrations of IL2, IL5, IL10 and interferon (IFN)-γ in the supernatant were then quantified by a sandwich ELISA specific to each cytokine (ELISA MAX™ Standard, BioLegend, San Diego, CA, USA) following the manufacturer’s instructions.

## Results

### Clinical symptoms of Japanese cedar pollinosis

**Fig. 2:**
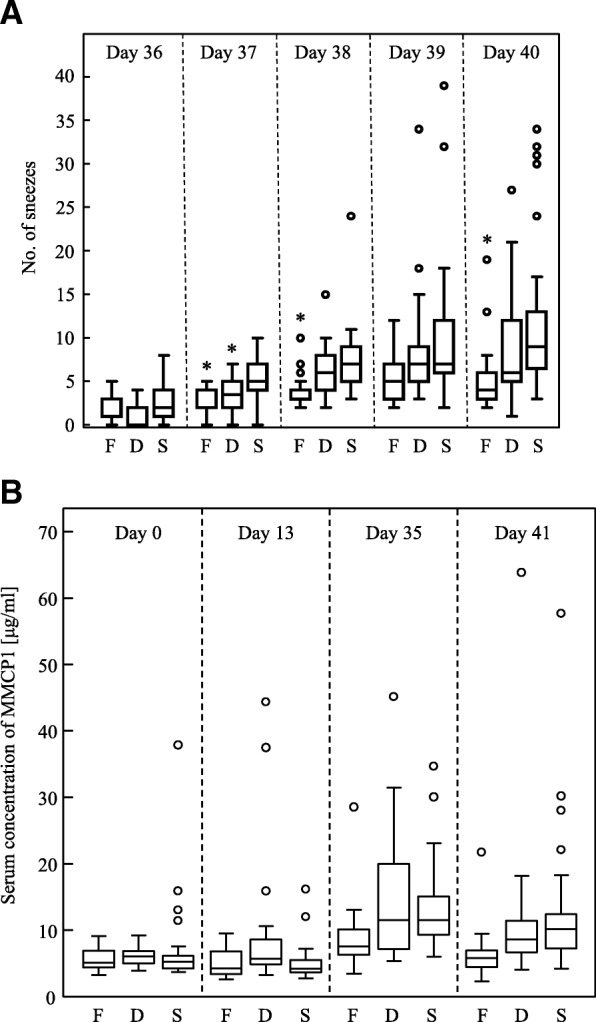
The number of sneezes and serum MMCP1 concentration for all mice in three independent experiments. **a** The number of sneezes during a 5-minute period soon after an intranasal SBP challenge were counted for mice treated with FPP (F, *n* = 16), 10-fold-diluted FPP (D, *n* = 16), and saline (S, *n* = 30). *p < 0.05 by non-repeated measures ANOVA with post hoc Dunnett test using saline as a negative control. **b** Serum MMCP1 concentration was analyzed for mice treated with FPP (F, *n* = 16), 10-fold-diluted FPP (D, *n* = 16), and saline (S, *n* = 30). Statistical significance was determined by nonrepeated ANOVA

To elucidate the capacity of FPP to attenuate clinical symptoms of JCP, we fed BALB/c mice 100 μL of FPP, 10-fold-diluted FPP, or saline every day for 40 days and sensitized the mice with Japanese cedar pollen allergens, i.e., SBP (Fig. 1). SBP is a fraction of ion-exchange chromatography of Japanese cedar pollen extract, and it consists of the two major Japanese cedar pollen allergens Cry j 1 and Cry j 2 [26]. In the present study, FPP- or saline-fed mice were intraperitoneally injected with SBP adsorbed on alum, a Th2-prone immune-adjuvant, followed by a subcutaneous injection of SBP 14 days later (Fig. 1). After the daily intranasal administration of SBP for five consecutive days, we counted the number of sneezes by each mouse during 5-minute period after the administration. The numbers of sneezes steadily increased over the five days in all treated mice, but the numbers were significantly lower in the FPP-treated mice (shown by F in Fig. 2a) compared to the numbers in the mice treated with 10-fold diluted FPP (D) and saline (S) on days 37, 38 and 40 (Fig. 2a). The numbers of sneezes in the mice treated with 10-fold-diluted FPP were significantly reduced compared to those in the mice treated with saline on day 37 and tended to be reduced compared to those treated with saline on days 38, 39, and 40, but without statistical significance (Fig. 2a). These data suggest that the consecutive oral intake of FPP potentiate to ameliorate nasal symptoms of JCP.

Supportively, serum level of mouse mast cell protease (MMCP)1 tended to be suppressed in FPP-treated mice than those in saline-treated mice after intranasal SBP-challenge (day 41) as a dose-dependent manner, although without statistical significance by nonrepeated ANOVA (Fig. 2b). In the two-group comparisons, the serum MMCP1 levels at day 35 and 41 were significantly different between the FPP-consumed (F) and saline-consumed (S) groups by the unpaired Student t test (*p* < 0.05). The serum MMCP1 level was comparable before immunization (day 0 and 13), but the level tended to be suppressed in mice consumed FPP (day 35 and 41) (Fig. 2b). These data suggest FPP-consumption may ameliorate nasal symptoms by suppressing mast cell degranulation.

### Antigen-specific immunoglobulin titers after FPP-treatment

**Fig. 3:**
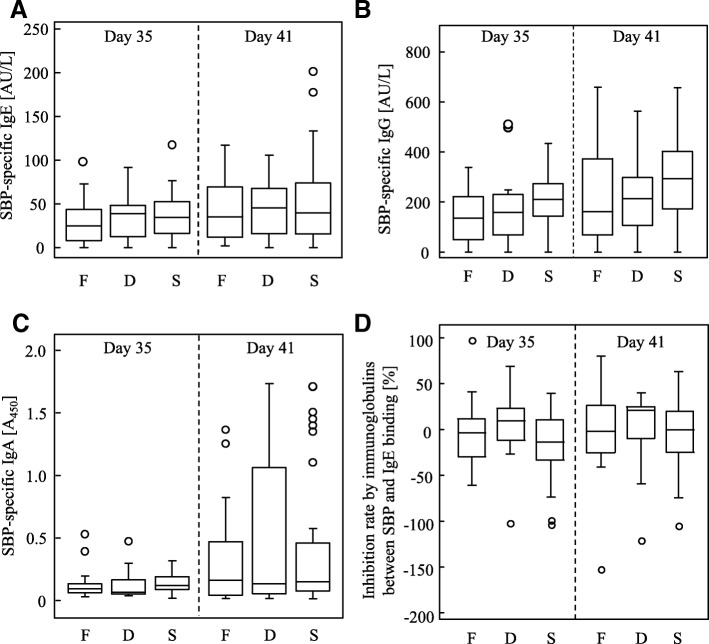
SBP-specific immunoglobulin titers for all mice in three independent experiments. **a** SBP-specific IgE titer of FPP-fed (F, *n* = 16), 10-fold-diluted FPP-fed (D, *n* = 16) and saline-fed (S, *n* = 30) mice at pre- (day 35) and post- (day 41) intranasal challenge with SBP. SBP-specific IgE titer for pooled sera from SBP-sensitized mice was used as standard serum and was determined as 1 kAU/L. Statistical significance was determined by nonrepeated ANOVA. **b** SBP-specific IgG titer of FPP-fed (F, *n* = 16), 10-fold-diluted FPP-fed (D, *n* = 16) and saline-fed (S, *n* = 30) mice at pre- (day 35) and post- (day 41) intranasal challenge with SBP. SBP-specific IgG titer for pooled sera from SBP-sensitized mice was used as standard serum and was determined as 1 kAU/L. Statistical significance was determined by nonrepeated ANOVA. **c** Intensity of SBP-specific IgA of FPP-fed (F, *n* = 16), 10-fold-diluted FPP-fed (D, *n* = 16) and saline-fed (S, *n* = 30) mice at pre- (day 35) and post- (day 41) intranasal challenge with SBP. Absorbance for 450 nm was plotted on the *y*-axis for each mouse after a colorimetric ELISA for SBP-specific IgA. The significance of differences was determined by nonrepeated ANOVA. **d** The inhibition of binding between SBP and serum IgE by serum immunoglobulins of FPP-fed (F, *n* = 16), 10-fold-diluted FPP-fed (D, *n* = 16) and saline-fed (S, *n* = 30) mice at pre- (day 35) and post- (day 41) intranasal challenge with SBP is shown as the inhibition rate. The significance of differences was determined by nonrepeated ANOVA

In the present study, the numbers of sneezes were significantly reduced in the FPP-treated mice, so we analyzed the serum SBP-specific antibody titers at pre- and post-intranasal SBP administration. The serum SBP-specific IgE antibody was undetectable before systemic immunization with SBP (day 0 and 13, data not shown). Unexpectedly, the SBP-specific IgE antibody titers after immunization were comparable among the FPP-, 10-fold-diluted FPP- and saline-treated mice even at post-intranasal SBP administration (day 41 in Fig. 3a).

We next analyzed the SBP-specific IgG and IgA titers in serum, because SBP-specific IgG and/or IgA contributes to the inhibition of binding between SBP and IgE which binds to high-affinity IgE receptors (FcɛRIs) on mast cells [1, 12]. Serum SBP-specific IgG and IgA antibodies were undetectable before sensitization (day 0 and day 13, data not shown). Neither the SBP-specific IgG titer nor the IgA titer differed significantly among the groups after systemic sensitization (day 35) and after intranasal challenge (day 41) (Fig. 3b, c). The binding between SBP and SBP-specific IgE was not inhibited by other subtypes of immunoglobulin including IgG and IgA as shown by an inhibition ELISA (Fig. 3d) [6, 28]. These data suggest that FPP reduces the numbers of sneezes independently with changes of antigen-specific immunoglobulin concentrations.

### T-cell activation after FPP treatment

**Fig. 4:**
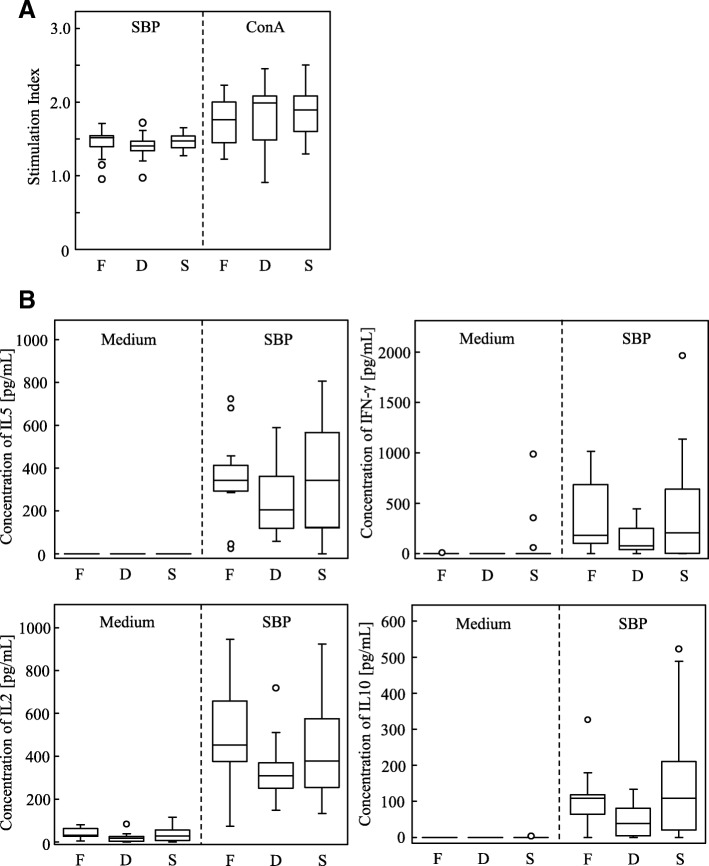
Proliferation and cytokine production of splenocytes after/without stimulation with SBP. **a** Proliferation of splenocytes from mice orally administered FPP (*n* = 11), 10-fold-diluted FPP (*n* = 11), or saline (*n* = 24) for all mice in two independent experiments. Murine splenocytes were cultured with/without SBP or Con A for 3 days, and BrdU was added and cultured for 18 h. The stimulation index was calculated for each mouse. Statistical significance was determined by nonrepeated ANOVA. **b** Cytokine production from each mouse orally administered FPP (*n* = 11), 10-fold-diluted FPP (*n* = 11), or saline (*n* = 24) for all mice stimulated with or without SBP for 3 days from two independent experiments. Statistical significance was determined by nonrepeated ANOVA

Interleukin (IL) and interferon (IFN) play crucial roles in the activation of antigen-specific T cells and inflammatory cells [29]. Here we analyzed immunological changes of splenocytes isolated from JCP model mice treated with FPP or saline. All of the SBP-sensitized JCP model mice showed positive proliferation in response to SBP stimulation in vitro (SBP in Fig. 4a). The stimulation indexes of the splenocytes stimulated with SBP or with Con A were comparable among the FPP-treated and saline-treated mice (Fig. 4a).

We then analyzed the cytokine profile from splenocytes after stimulation with SBP. We quantified IL5, a Th2-type cytokine; IFN-γ, a Th1-type cytokine; IL2, an important cytokine for T-cell proliferation and the induction of anergy; and IL10, a regulatory cytokine from Th2 cells and Tregs [1, 29]. The concentrations of all cytokines tested in culture supernatant tended to be lower in those from the 10-fold-diluted FPP-treated mice, but there was no significant difference among the FPP-, 10-fold-diluted FPP-, and saline-treated mice (Fig. 4b). These data demonstrate that FPP attenuated the sneezing by JCP mice without changing the systemic immune balance.

## Discussion

Our findings clarified that the intake of FPP has the effect of attenuating the symptoms of JCP without changing systemic immunological parameters in a JCP mouse model. We speculate that FPP or its metabolite may act on mast cells directly or indirectly to suppress mast cell degranulation on nasal mucosa, resulting in decreased numbers of sneezes in FPP-administered mice. It is interesting to investigate whether FPP can prevent the degranulation of mast cells in vitro, but because of its high viscosity and food-fiber-rich component, it is difficult to examine FPP’s anti-degranulation effects on mast cell in vitro. Further studies are needed to characterize the anti-allergic component(s) after the segregation of components of FPP by solvent fractionation and chromatography.

In a prophylactic model of JCP used in this study, the SBP-specific IgG, IgA, and IgE titers were undetectable before systemic sensitization with SBP (day 0 and 14, data not shown). However, levels of the serum SBP-specific immunoglobulins were significantly and similarly increased in all groups after systemic immunization and intranasal challenge with SBP (Fig. 3a-c). These data suggest that consumption of FPP does not affect the development of antigen-specific B cell or class-switch recombination of the B cell.

Antigen-specific IgG acts as an inhibitor for allergic symptoms by competing with IgE for binding to allergens [1]. In the present study, serum SBP-specific IgA and IgG antibodies did not significantly prevent binding between SBP and serum SBP-specific IgE antibody by ELISA inhibition assay (Fig. 3d). However, it is possible that SBP-specific IgA antibody prevents binding between SBP and SBP-specific IgE antibody in nasal mucosa and attenuates following sneezing reaction after intranasal challenge with SBP. To analyze the level of SBP-specific IgA antibody and numbers of IgA-producing B cell in nasal mucosa would be important to elucidate precise mechanisms of FPP to ameliorate nasal symptoms in the future studies.

In the present study, we analyzed numbers of sneezing as a primary parameter for clinical symptoms for pollinosis (Fig. 2a). Numbers of nasal rubbing after nasal allergen provocation is another important parameter for clinical symptoms of allergic rhinitis. Numbers of nasal rubbing may correlate with numbers of sneezes, albeit the background number is higher in nasal rubbing than in sneezes in unsensitized mice [30, 31]. We tried to count nasal rubbing after intranasal challenge of SBP, but those counts were quite lower than those sensitized with ovalbumin (OVA), a gold-standard allergen. So, we could not select the rubbing behavior as a readout, and selected sneezing as a reliable clinical parameter for JCP. The amount of SBP used in the present study may not enough to induce stable nasal rubbing, because 100 or 500 μg Japanese cedar pollen grains was used to induce nasal rubbing as a clinical parameter in other studies [31, 32]. Much higher dose of SBP would be needed for intranasal administration to induce stable nasal rubbing as a reliable clinical parameter for JCP. Analysis of other parameters for clinical symptoms, such as nasal rubbing, histamine release from peripheral blood cells, and histamine or β-hexosaminidase concentration in nasal lavage fluid, would support the capacity of FPP-consumption to attenuate JCP symptoms. Other clinical parameters such as rhinoconjunctivitis or conjunctivitis after nasal or ocular allergen challenge may also support FPP’s potential to prevent JCP symptoms.

The average and median numbers of sneezes at day 40 were 4 and 5.7 in FPP-consumed mice and 9 and 11.9 in saline-treated mice (Fig. 2a). The suppression of sneezes by FPP consumption was mild, but the numbers were reduced in FPP-consumed mice as half as those in saline-treated mice with statistical significance. The level of suppression in sneezing is similar degree to those by intranasal treatment of anti-Cry j 1 monoclonal antibody Fab fragment, which suppresses mast cell degranulation by prohibit binding between Cry j 1 and anti-Cry j 1 IgE antibody on mast cell [31]. Although we found mild attenuation of sneezing with statistical significance by FPP-consumption in mice, a double-blinded, placebo-controlled, randomized clinical trial will be needed to clarify the preventive effects of FPP-consumption on JCP in human.

In this study, we administered 0.1 ml of FPP (138 mg) once daily to mice (calculated as 5.5 g/kg for FPP and 0.55 g/kg for 10-fold-diluted FPP). Dose of FPP for human consumption will be calculated as daily 199 ml (275 g). The volume seems too much for daily consumption as a supplemental nutrient food. A phase II clinical trial is needed to determine the effective and optimal dose of FPP to prevent clinical symptom of JCP in human.

Clinical studies will be needed to determine optimal dose of FPP and to elucidate FPP’s capacity to attenuate JCP symptoms in human. In the present preliminary study, we suggest potential of crude FPP to attenuate sneezing without changing systemic immunological balance in mice (Fig. 2a, 3, and 4). We should determine the active ingredient(s) in FPP to attenuate JCP symptoms and elucidate anti-degranulation capacity of the active ingredient(s) using mast cell in vitro. Amelioration of sneezing, nasal rubbing and suppression of mast cell degranulation by the ingredient(s) have to be analyzed in preclinical animal studies before proceeding to human clinical studies.

Our previous study of 8-hydroxy-5,7-dimethoxyflavanone isolated from *Perilla frutescens* showed its capacity to suppress the degranulation of mast cells directly in vitro and to suppress sneezing after an intranasal administration of Japanese cedar pollen extract in a JCP mouse model, without affecting the serum titer of Japanese cedar pollen allergen-specific IgE and IgG titers [10]. Another study demonstrated that the oral intake of sake lees fermented with lactic acid bacteria attenuated OVA-induced allergic rhinitis, without significant impact on OVA-specific immunoglobulin titers [33]. The fermented sake lees also directly inhibit the degranulation of mast cells in vitro [33]. These studies demonstrate that consumption of food-derived anti-allergic ingredient(s) can suppress allergic rhinitis without changing systemic immunological parameters such as serum allergen-specific immunoglobulin titers and cytokine profile. Although serum level of MMCP1 was not analyzed in the studies, the food-derived ingredient(s) suppresses lgE-dependent degranulation of RBL-2H3 bosophilic cell in vitro. These studies suggest that the consumption of food-derived anti-allergic ingredient(s) attenuates symptoms of JCP by suppression of allergen-specific mast cell degranulation in vivo.

In the present study, FPP-intake tended to suppress serum level of MMCP1, a serum marker for mucosal mast cell degranulation, after SBP-immunization at day 41, although without the statistical significance by nonrepeated ANOVA (Fig. 2b). The data implicate the possibility that FPP-consumption could prevent mast cell degranulation in vivo. High anti-oxidant capacity of FPP was reported in rodent and in Japanese flounder [20, 22, 24]. Recent finding showed mitochondria-targeted anti-oxidant inhibited mast cell degranulation in vivo and in vitro [34]. Another study also showed jacareubin isolated from the heartwood of the tropical tree, *Callophyllum brasilense*, inhibited IgE-induced mast cell degranulation by its high anti-oxidant capacity [35]. Jacareubin inhibited calcium influx into mast cells accompanied with a blockage on the accumulation of the reactive oxygen species (ROS) in mast cells. These reports implicate FPP’s high anti-oxidant capacity may contribute for reduction of the sneezing by inhibiting accumulation of ROS. Analysis of the capacity of FPP’s component(s) to inhibit ROS accumulation in mast cells could be important to elucidate precise mechanisms how FPP ameliorates clinical symptom of JCP.

Another possible mechanism to ameliorate the clinical symptoms of JCP by the consumption of FPP without changing the systemic immune balance may be ascribed to changes in microbiota in the intestine and/or the gut environment. It was reported that the oral intake of mixture of *Lactococcus lactis* KF140, *Pediococcus pentosaceus* KF159, *Lactobacillus pentosus* KF340, *Lactobacillus paracasei* 697 and *Bacillus amyloliquefaciens* 26 N reduced OVA-induced food allergy with a suppression of total IgE, OVA-specific IgE, Th2-type cytokine production, and IL17 production [36]. Aside from food allergy, the consumption of a probiotic combination of *Lactobacillus acidophilus* NCFM and *Bifidobacterium lactis* BI-04 ameliorated the nasal symptoms in birch pollinosis patients with an increase in the fecal IgA titer [37]. The probiotic combination also prevented the birch pollen-induced infiltration of eosinophils in nasal mucosa.

These reports indicate that a component of FPP or FPP’s metabolites may suppress the activation and accumulation of inflammatory cells in nasal mucosa after a nasal allergen challenge with SBP, although it was shown that the intake of FPP did not influence the populations of *Bifidobacterium*, *Lactobacillus*, *Bacteroides*, *Prevotella* or *Clostridium* in the cecum in rats [38]. The changes in gut microbiota brought about by FPP consumption and its contribution to the attenuation of JCP rhinitis should be clarified in the near future.

## Conclusions

FPP has the potential to attenuate clinical symptoms of JCP without changing systemic immunological properties. Our present findings suggest that FPP or its metabolite may suppress mast cell degranulation or accumulation directly or indirectly at an inflammatory locus. Further studies are needed to clarify the anti-allergic mechanisms underlying the effects of FPP consumption.

## Abbreviations

FPP: Fermented plant product
IFN: Interferon
Ig: Immunoglobulin
IL: Interleukin
JCP: Japanese cedar pollinosis
OVA: Ovalbumin
SBP: Sugi basic protein
